# Oseltamivir for treatment and prevention of pandemic influenza A/H1N1 virus infection in households, Milwaukee, 2009

**DOI:** 10.1186/1471-2334-10-211

**Published:** 2010-07-20

**Authors:** Edward Goldstein, Benjamin J Cowling, Justin J O'Hagan, Leon Danon, Vicky J Fang, Angela Hagy, Joel C Miller, David Reshef, James Robins, Paul Biedrzycki, Marc Lipsitch

**Affiliations:** 1 Center for Communicable Disease Dynamics, Department of Epidemiology, Harvard School of Public Health, Boston MA, USA; 2 School of Public Health, The University of Hong Kong, Hong Kong, China; 3 Mathematics Institute and the Department of Biology, Warwick University, Coventry, UK; 4 Milwaukee Health Department, Milwaukee WI, USA; 5 University of Oxford, Oxford, UK; 6 Fogarty International Center, National Institutes of Health, Bethesda, Maryland 20892, USA

## Abstract

**Background:**

During an influenza pandemic, a substantial proportion of transmission is thought to occur in households. We used data on influenza progression in individuals and their contacts collected by the City of Milwaukee Health Department (MHD) to study the transmission of pandemic influenza A/H1N1 virus in 362 households in Milwaukee, WI, and the effects of oseltamivir treatment and chemoprophylaxis.

**Methods:**

135 households had chronological information on symptoms and oseltamivir usage for all household members. The effect of oseltamivir treatment and other factors on the household secondary attack rate was estimated using univariate and multivariate logistic regression with households as the unit of analysis. The effect of oseltamivir treatment and other factors on the individual secondary attack rate was estimated using univariate and multivariate logistic regression with individual household contacts as the unit of analysis, and a generalized estimating equations approach was used to fit the model to allow for clustering within households.

**Results:**

Oseltamivir index treatment on onset day or the following day (early treatment) was associated with a 42% reduction (OR: 0.58, 95% CI: 0.19, 1.73) in the odds of one or more secondary infections in a household and a 50% reduction (OR: 0.5, 95% CI: 0.17, 1.46) in the odds of a secondary infection in individual contacts. The confidence bounds are wide due to a small sample of households with early oseltamivir index usage - in 29 such households, 5 had a secondary attack. Younger household contacts were at higher risk of infection (OR: 2.79, 95% CI: 1.50-5.20).

**Conclusions:**

Early oseltamivir treatment may be beneficial in preventing H1N1pdm influenza transmission; this may have relevance to future control measures for influenza pandemics. Larger randomized trials are needed to confirm this finding statistically.

## Background

With the advent of the influenza season in the Northern hemisphere, various measures to control the spread of an epidemic and to reduce severe morbidity and mortality are being utilized. One type of such control measures is the usage of antiviral drugs, specifically the neuraminidase inhibitors oseltamivir and zanamivir. Recent CDC guidelines http://www.cdc.gov/h1n1flu/antiviral.htm emphasize antiviral drug usage for severe cases as well as for high risk individuals. As the 2009 H1N1 pandemic has shown, large scale vaccine distribution in many countries may occur only after an epidemic has peaked; under these circumstances, the appropriate use of a limited supply of antivirals becomes even more paramount.

The ability of oseltamivir, particularly if taken during the earlier stages of influenza infection, to alleviate symptoms and shorten their duration is well documented for seasonal influenza [1-3]. For H1N1pdm infections, two recent studies suggest that early oseltamivir treatment for hospitalized H1N1 patients was beneficial in reducing the risk of death [4] and ICU admission [5]. In [6] a subset of the present authors consider the net benefits of pre-dispensing antivirals to high-risk individuals during an influenza pandemic, where the measure of the benefit is the number of lives saved by antivirals in the whole population. One factor which makes pre-dispensing beneficial is that individuals to whom antivirals have been pre-dispensed may be able to initiate treatment early, reducing their risk of progression to severe disease. In this paper we study another benefit of early oseltamivir treatment, namely its potential role in preventing influenza transmission to others [7].

One setting where influenza transmission can be studied is within households. This is particularly valuable in the early stages of an epidemic, where multiple out-of-household infections are rare, and subsequent (secondary) cases can be reasonably attributed to the first case in a household (the index), and possibly to other secondary cases. In this context, we studied the relation between the timing of oseltamivir usage for the index case and the existence of secondary case(s) in a household. Our study is based on the data gathered by the City of Milwaukee Health Department, where extensive efforts on epidemiological tracing of confirmed cases and their contacts made it possible to recover the chronology of symptom onsets and oseltamivir usage in a number of households.

The main question we sought to address is whether early index oseltamivir usage (on onset day or the following day) has benefits in reducing the risk of transmission to other household members (and thus likely also to other individuals outside the household). Data shows that indeed such benefit is noticeable. However the confidence bounds are wide and the conclusions are not statistically significant due to a small sample of households where early index oseltamivir usage took place; we also discuss the effect of potential biases and our attempts at addressing them. Results similar to ours with a statistically significant conclusion for a larger study of seasonal influenza appear in [8].

## Methods

### Sources of data

Epidemic data gathered by the Milwaukee Health Department between mid April to Mid June 2009 was used in the study. Initially cases were concentrated on or near the south side of Milwaukee which has a large Hispanic population, however in a matter of weeks the epidemic had spread throughout the city.

Data on influenza progression in individuals and their contacts were collected by the City of Milwaukee Health Department (MHD), Division of Disease Control and Environmental Health. Cases with confirmed pandemic influenza H1N1 infection by RT-PCR were entered into a centralized database. Cases were then assigned to public health nurses for public health case management. Public health case management consisted of completion of the MHD H1N1 surveillance form, identification of possible source and spread of disease, intervention to prevent additional spread as well as general health and hygiene education. Case monitoring was done through phone interviews; generally 1-2 phone contacts with households (beyond the initial detection) took place. Only households where the last phone interview occurred at least a week after the index onset were included in the study.

Individual case report/surveillance forms filled out by the nurses had demographic information, information on confirmation status, presence of influenza-like illness (ILI), listing of symptoms/onset date, antiviral/antibiotic usage etc, as well as (generally less complete) information on individual's contacts; initial information was updated through phone contacts. Case report forms were grouped by households, with some members having an individual form, while others (possibly symptomatic) not having one.

Data from case report forms from 362 households were entered electronically. The primary analysis for the oseltamivir effect is based on the 135 of the above 362 households that fulfilled the following criteria a)-d).

a) Symptom onset dates for all household members were listed.

b) The primary (index) case in a household had a laboratory-confirmed infection and an individual case report form with contacts listed, and the case report form(s) for the household members contained no contradictions (e.g. two case report forms listing the same household with different information).

c) The household had a follow-up period of at least 7 days since the index symptom onset.

d) Start date for oseltamivir usage by the index (if any), and the start date of any secondary oseltamivir usage in a household could be ascertained.

Contacts were considered infected if they were either confirmed, or had ILI (fever + cough or sore throat), or had at least two symptoms listed. Fever in cases possessing a case report form had a temperature reading for the majority of cases; fever in symptomatic cases frequently had no temperature reading next to it. A contact with only one symptom or with only the "symptoms" box checked disqualified the household from being used for the primary analysis unless the onset date for that contact was given and it was no earlier than the first secondary onset in a household (3 such contacts with one symptom each appear in the primary analysis cohort).

To increase efficiency of data entry in terms of assessing the effect of early oseltamivir usage, some of the cases for the primary analysis were entered into a database from paper forms *only *if they met criteria a)-d). This leads to no bias for the selection of the primary analysis cohort as **all **the households obeying criteria a)-d) among the first several hundred households (in chronological order) made it to the primary analysis cohort. We recognized that some of the selection criteria a)-d) may bias estimates of other quantities we were interested in, such as the time between the index onset and the first secondary infection in a household. Thus, in addition to households screened for obeying criteria a)-d), we entered data from a number of households which appeared in chronological order without checking whether they obey any criteria. The distribution of times to first secondary case in the household was assessed from 128 of these households - those households were selected to obey criteria a)-b) but not necessarily criteria c)-d) which may bias the latter distribution. The first secondary infection was used because of the lack of information on the subsequent path of transmission, such that it was uncertain whether each non-index infection represented a secondary or tertiary or further infection.

### Statistical Analysis

Household characteristics were described with means and standard deviations, and medians and inter-quartile ranges. Secondary attack rates at the household level were estimated as the proportion of households with one or more secondary cases. Secondary attack rates at the individual level were estimated as the proportion of household contacts who were infected (based on the definition of infection described above).

The effect of oseltamivir treatment and other factors on the household secondary attack rate was estimated using univariate and multivariate logistic regression with households as the unit of analysis.

The effect of oseltamivir treatment and other factors on the individual secondary attack rate was estimated using univariate and multivariate logistic regression with individual household contacts as the unit of analysis, and a generalized estimating equations approach (robust variance estimator) was used to fit the model to allow for clustering within households [9]. For 21 contacts with unknown age, multivariable ORs were adjusted via multiple imputations.

We estimated 95% confidence intervals for secondary attack rates using the exact binomial method at the household level, and the cluster bootstrap method [10] at the individual level.

Statistical analyses were performed using R version 2.8.0 (R Development Core Team, Vienna, Austria).

### Ethics statement

Our study, which utilized anonymized data on H1N1 cases and their household contacts collected by the Milwaukee Health department was performed according to the established guidelines. Our work was reviewed by the Human Subjects Committee at the Harvard School of Public Health. Because the research involved existing data recorded in such a manner that subjects could not be identified, it was designated "not human subjects" (protocol # 17770-101).

## Results

### Timing of oseltamivir usage and transmission: household level

The timing of oseltamivir usage by index cases is shown in Table 1. There is a monotonic increase in the probability of secondary attack with the time from symptom onset to oseltamivir initiation by the index. Interestingly, never-users of oseltamivir are less likely to have secondary cases in the household than those who used oseltamivir on day 2 or day 3. A candidate explanation for this finding is that it reflects reverse causality; index cases who had a secondary case in their household were more likely to receive oseltamivir, perhaps because they were more likely to seek treatment. Consistent with this hypothesis, of the 19 households with a secondary attack where the index took oseltamivir on or after day 3, 12 had the index taking oseltamivir after the day of the secondary attack, and 3 more took it on the day of the secondary attack, with only 4 indices starting it before secondary symptoms.

**Table 1:** Index oseltamivir start day and HH size vs. secondary attack

Day of index oseltamivir (onset = 0)	Total number of households	Secondary attack	Percent
0	8	1	12.5%

1	21	4	19.0%

2	11	3	27.3%

3+	51	19	37.3%

Never	44	10	22.7%

**HH size**			

2-3	48	7	14.6%

4	41	14	34.1%

5+	46	16	34.8%

Table 1 also shows the relationship between household size and the probability of at least one secondary case in the household, with an increasing risk of a secondary attack with household size.

Tables 2 summarizes the results of statistical analysis of the effect of the timing of index oseltamivir usage, household size and the age of the index on the existence of a secondary attack in a household. Oseltamivir use on the day of symptom onset or the day after was associated with a 42% reduction in the odds of a secondary case in the household compared to use on day 3 or later or never; this effect is close to the null for use on day 2, and the confidence intervals for both estimates include 1. Larger household size was associated with a higher risk of transmission, though the multivariate logistic regression confidence intervals for the relative risk compared to household sizes 2-3 include 1. Index age showed no association with the risk of secondary transmission

**Table 2:** Probabilities of at least one secondary case in the household

	n	Any secondary infections in household					
		
		SAR	(95% CI)	Univariate OR	(95% CI)	Multivariate OR	(95% CI)
*Index oseltamivi usage from his/her onset*							
Within 1 day	29	0.17	(0.06, 0.36)	0.47	(0.16, 1.37)	0.58	(0.19, 1.75)
1-2 days	11	0.27	(0.06, 0.61)	0.85	(0.21, 3.45)	1.15	(0.27, 4.99)
3+ days/No usage	95	0.31	(0.21, 0.41)	-		-	
							
Household with adult index	37	0.19	(0.08, 0.35)	-		-	
Household with child index	98	0.31	(0.22, 0.41)	1.89	(0.75, 4.78)	1.30	(0.47, 3.57)
							
Household size (# of ppl)							
≤ 3	48	0.15	(0.06, 0.28)	1.00		-	
4	41	0.34	(0.20, 0.51)	3.04	(1.09, 8.50)	2.81	(0.98, 8.10)
≥ 5	46	0.35	(0.21, 0.50)	3.12	(1.14, 8.54)	2.73	(0.94, 7.88)

### Factors associated with transmission at the individual level

Table 3 shows the relation between the ages of the indices and their (infected/uninfected) contacts. Each row lists the index's age (child/adult). The columns list the total numbers of household contacts of a certain age group that all such indices have, and how many of them were infected. At the individual level we identified infections in 55/411 household contacts, corresponding to a secondary attack rate of 13.4%. Table 4 summarizes the results of statistical analysis of the effect of the timing of index oseltamivir usage, household size and the ages of the index and contacts on H1N1 influenza transmission at the individual level. Oseltamivir use on the day of symptom onset or the day after was associated with a 50% reduction in the odds of a secondary case in the household compared to use on day 3 or later or never; this effect is attenuated for use on day 2, and the confidence intervals for these estimates include 1. Younger contact age was associated with a higher risk of infection, while household size showed no association with individual risk of transmission.

**Table 3:** Ages and secondary cases

	Infected child/contact child		Infected adult/contact adult		Infected unknown/contact unknown	
	**n/n**	**%**	**n/n**	**%**	**n/n**	**%**

**Child index**	30/133	22.6%	15/176	8.5%	2/19	10.5%

**Adult index**	4/40	10%	4/41	9.8%	0/2	

**Table 4:** Secondary attack rates at the individual level

	n	Secondary infection in household contacts					
		
		SAR	(95% CI)	Univariate OR	(95% CI)	Multivariate OR	(95% CI)
*Index oseltamivir usage from his/her onset*							
Within 1 day	29	0.09	(0.03, 0.17)	0.53	(0.20, 1.37)	0.57	(0.20, 1.62)
1-2 days	11	0.17	(0.00,0.33)	1.06	(0.30, 3.69)	1.11	(0.30, 4.11)
3+ days/No usage	95	0.14	(0.10, 0.19)	-		-	
							
Household with adult index	37	0.10	(0.04, 0.17)	1.00		1.00	
Household with child index	98	0.14	(0.10, 0.19)	1.48	(0.62, 3.51)	1.55	(0.58, 4.15)
							
Adult contact*	217	0.09	(0.05, 0.13)	-		-	
Child contact*	173	0.20	(0.13, 0.27)	2.71	(1.47, 5.02)	2.79	(1.50, 5.20)
							
Household size (# of ppl)							
≤ 3	48	0.11	(0.04, 0.20)	-		-	
4	41	0.14	(0.07, 0.20)	1.26	(0.48, 3.30)	0.93	(0.32, 2.72)
≥ 5	46	0.14	(0.08, 0.20)	1.25	(0.48, 3.24)	0.74	(0.26, 2.10)

* Multivariable ORs adjusted for unknown age group for 21 contacts via multiple imputations with 10 imputations

### Time from index onset to first secondary infection

**Figure 1:**
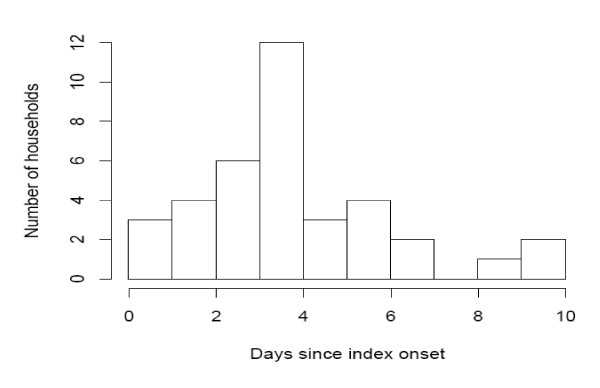
**Distribution of times from index onset to a first secondary onset in a household**.

We studied the distribution of time from index onset to a first secondary onset (FSO) in a household. Because of the clustering of susceptibles, this distribution is inherently shorter than the individual infectiousness profile distribution, or a serial interval distribution in a mass action model. One household with FSO > 10 (17) was discarded as such a long FSO is likely to reflect a transmission from outside the household. The mean (sd) FSO was 3.32 (2.23). The distribution is plotted in Figure 1.

## Discussion

Our analysis of data from City of Milwaukee households supports the following conclusions: 1) Early oseltamivir use was associated with approximately a 42% reduction in the odds of at least one secondary case in the household, though this finding was not statistically significant and may be affected by some of the biases described below 2) Children had a statistically significantly higher risk of becoming infected than adults. 3) Household size greater than 3 was associated with an increased risk of at least one secondary case in the household, though this effect was statistically significant only in the univariate analysis and could be impacted by a selection bias. No association between individual risk of a symptomatic secondary infection and household size was found.

Observational studies of household transmission are crucial for rapid evidence-gathering in a newly emerging influenza pandemic, but they are also susceptible to a number of biases intrinsic to observational studies, compounded by the sometimes formidable logistical challenges of gathering accurate data during a local public health response to a pandemic. We shall now present several potential sources of biases and our attempts at addressing them.

A key potential source of bias is a form of selection bias [11], related to the fact that not all infected households were recorded by the City of Milwaukee Health Department, since not all infected persons came to medical attention and received testing. One might expect that some households would come to medical attention only after having secondary cases in the household, and that this may have occurred several days after the onset of symptoms in the index. Such households would by definition have a secondary case but no early oseltamivir use (since the index case was not initially diagnosed). We have attempted to correct for this form of bias by excluding households for which the index case did not have a case report form, since these would be households in which the secondary cases would be most likely to have triggered the inclusion of the household in the study. As expected, this moved the univariate odds ratio for early oseltamivir use and secondary transmission toward the null value of 1, from 0.28 to 0.47. Additionally this selection bias should correlate with a larger household size. Since household size was included in the multivariate analysis on the household level and was found to be positively associated with having at least one secondary case (see Table 2), this should further reduce the bias.

Another potential source of bias is related to the inclusion criteria for data completeness in the primary cohort which have differentially excluded households with different outcomes. For instance, oseltamivir receipt with a missing date or a missing date for symptom onset could cause exclusion from the primary analysis cohort. Although our study is a cohort study, this form of selection bias is analogous to that present in all case-control studies, in which the outcome influences the probability of inclusion in the study; however this alone does not bias the odds ratio. To see that, consider the odds of inclusion into the primary analysis cohort for households with vs. without secondary attack given index oseltamivir, and the corresponding odds given no index oseltamivir. It is a reasonable first approximation that those 2 odds (2 ratios of probabilities) are the same, because the probability that index oseltamivir start date is recorded should not differ depending on whether there was a secondary case in the household. Hence, in this approximation, the odds ratio for inclusion is 1, or, in other words, the odds ratios in the original population and in the group analyzed in this study are the same.

The associations between early oseltamivir usage and reduced risk of secondary attack seen in univariate analyses were attenuated slightly in multivariate analysis. This attenuation reflects a positive correlation between smaller household sizes (which are also protective) and early index oseltamivir usage in the data.

We do not believe that oseltamivir chemoprophylaxis contributed to a significant upward bias in our estimate of the benefit of early oseltamivir index usage. In fact, out of the 24 households with index oseltamivir usage on days 0-1 and no secondary attack, only 2 had some contact chemoprophylaxis.

We found that the time from index onset to the onset of the first secondary case in the household had a mean of 3.32 days and a standard deviation of 2.23 days, broadly consistent with some previous estimates [12,13] and slightly longer than some other estimates for the serial interval for H1N1 [14-16]. Moreover the serial interval estimated from the first secondary case in this way will be somewhat shorter than the mean time at which a case will cause secondary infections [17], but this bias is relatively minor as there a rarely more than two cases in a household.

We have chosen a case definition for secondary cases that is slightly different from the Centers for Disease Control and Prevention surveillance definition of influenza-like illness (ILI) as fever plus cough or sore throat. A similar distinction was made in [14,18], both of which made estimates of the secondary attack rates for acute respiratory infection similar to our estimate. Influenza A/H1N1 occasionally leads to mild self-limiting illness, and not all the confirmed index cases had ILI. 9 of 55 contacts in the primary analysis cohort who met our definition of secondary cases failed to meet the standard ILI definition, and 3 of these individuals were tested and virologically confirmed.

In our sample, 21% (29/135) of index cases received oseltamivir within the first two days of symptoms. This likely represents an upper bound on the true frequency within the population, since inclusion in our study depends on coming to medical attention. It is therefore likely that little impact of oseltamivir treatment on total transmission of influenza occurred during the spring in Milwaukee.

Oseltamivir is thought to be effective in reducing transmission of seasonal influenza in households and other settings [3,7,8]. Our findings suggest that it is comparably effective in the setting of H1N1pdm infection in households. Given the biases inherent in observational studies conducted in the middle of an emerging pandemic, randomized studies are needed to assess the magnitude of this effect more precisely. The United States has limited supplies of oseltamivir, approximately enough for ¼ of the population to obtain one course. While this is larger than the supply of virtually any developing country, it is less than the stockpile held by some other wealthy countries, and less than most models predict would be necessary to undertake a sustained effort to reduce transmission with antiviral treatment [19] or chemoprophylaxis. In countries with a larger stockpile, these findings may support the consideration for use of treatment as a transmission-reduction measure, but the findings also suggest that early treatment of symptomatic cases is essential if transmission-reduction is to succeed. Given that the benefits of oseltamivir for the treated patient are best documented (for seasonal influenza) when treatment is given within 48 hours of symptom onset (though later treatment can also be beneficial, [20]), the results here, combined with considerations of individual benefit of early treatment in reducing disease severity [4,5], suggest that additional efforts are needed to ensure timely access to antiviral drugs.

## Conclusions

Early oseltamivir treatment may be beneficial in preventing H1N1pdm influenza transmission; this may have relevance to future control measures for influenza pandemics. Larger randomized trials are needed to confirm this finding statistically and to overcome possible biases.

## Competing interests

ML discloses consulting fees from the Avian/Pandemic Flu Registry (Outcome Sciences), funded in part by Roche, and from Novartis Vaccines and Diagnostics. All other authors declare no competing interests.

## Authors' contributions

A.H. and P.B. have collected the data. B.C., J.R., M.L., V.F. and E.G. have designed the study. V.F, B.C., J.J.O., J.C.M., D.R., L.D., M.L and E.G. have analyzed the data. E.G and M.L. wrote the manuscript. All authors read and approved the final manuscript.

## Pre-publication history

The pre-publication history for this paper can be accessed here:

http://www.biomedcentral.com/1471-2334/10/211/prepub
